# Profiling the diets of Classic Maya communities living in Southeastern Petén through stable isotope analysis

**DOI:** 10.1371/journal.pone.0353029

**Published:** 2026-07-22

**Authors:** Gloria Ivonne Hernández Bolio, Nadia Neff, Keith Prufer, Patricia Quintana, Viorel Atudorei, Mara Reyes, Lilian Corzo, Vera Tiesler

**Affiliations:** 1 Departamento de Física Aplicada, Centro de Investigación y de Estudios Avanzados del Instituto Politécnico Nacional, Unidad Mérida, Mérida, Mexico; 2 Center for Stable Isotopes, University of New Mexico, Albuquerque, New Mexico, United States of America; 3 Centro Universitario de Petén, Universidad de San Carlos de Guatemala, Flores, Guatemala; 4 Facultad de Ciencias Antropológicas, Universidad Autónoma de Yucatán, Mérida, Mexico; Universidad de Sevilla, SPAIN

## Abstract

During the Early and Late Classic periods (250–900 CE), Maya communities in southeastern Petén experienced rapid demographic growth, and organized themselves here not as centralized city states but as a network of complex, small-scale polities. As documented by the Archaeological Atlas of Guatemala (IDAEH), this area encompasses more than 400 registered sites across the northwestern Maya Mountains, several river basins, and humid savannas, and therefore offers a diversified ecological and cultural setting for exploring local subsistence strategies of the era. Excavation and sampling strategies targeted non-elite burials, providing a unique opportunity to reconstruct the diets of commoner populations on a regional scale, especially given that no prior isotopic research has been conducted in this vast area. For this study, we analyzed stable carbon (δ^13^C) and nitrogen (δ^15^N) isotopes from human bone collagen and δ^13^C from dental enamel carbonate to evaluate collective food selection, composition, and variability over time, as well as potential social or environmental influences. We contextualized these with the isotopic profiles in published research that covers the more hierarchically organized urban centers from other parts of the Maya area. Our own results reveal consistent maize-based diets that were likely supplemented by other plant-based foods and terrestrial animal resources, with inter-site differences reflecting local social and ecological contexts. Variation in isotopic values by age and the presence of skeletal modifications suggest that life-history and biocultural factors shaped dietary patterns. Overall, our findings demonstrate an increasing reliance on maize towards the end of the Classic period and even beyond the hegemonial collapse. Taken together, our findings in southeastern Petén’s populations expand our understanding of subsistence diversity and diet among non-elite Classic Maya populations.

## Introduction

Environmental and social conditions greatly influenced the foods consumed by ancient populations. Factors such as climate, ecosystem type, and hydrology largely determined the availability of resources, while local variation in diet was further shaped by age, sex, socioeconomic status, and regional identity [1–3]. At the same time, diet also reflected social, economic, and political circumstances. Therefore, reconstructing ancient foodways provides valuable insights into how past populations adapted to environmental constraints and managed periods of social and demographic change [4,5]. For the Classic-period Central Maya Lowlands, subsistence studies have focused mainly on major urban centers and monumental sites. These investigations have provided key information on long-term dietary changes and community responses to significant transitions associated with the Terminal Classic (750/800–900 CE) and Late Preclassic (300 BCE – 300 CE) periods [6]. However, this emphasis on highly urbanized sites and the predominance of elite individuals within their samples has in practice biased the overall populational view of Classic Maya subsistence patterns. Smaller and non-elite communities’ everyday strategies and adaptations remain comparatively understudied, despite constituting a fundamental proportion of the regional demography and actively contributing to the economy of Classic Maya society.

The communities of southeastern Petén represent small-scale political entities. Their geographic delimitations were often fluid and indeterminate, in contrast to the more centralized polities of northern Petén [7]. Although these centers mirrored the administrative and ritual structures of larger urban centers, they operated on a localized scale, with more equal access to natural and politically and economically regulated resources. Their areas of influence often overlapped and changed over time, reflecting dynamic patterns of resource sharing and interaction. This region flourished during the Early (300–600 CE) and Late Classic (600–750/800 CE) periods, reaching its peak in the Terminal Classic. Evidence of later Postclassic (900–1520 CE) occupation is present at some centers, while others appear to have been abandoned during the ninth and tenth centuries CE.

Within this context, we address several bioarchaeological questions: 1) Were the foodways of southeastern Petén populations distinct from those of the larger, centralized, and highly urbanized Maya cities? 2) Did all communities consume similar foods in comparable proportions? 3) Are isotopic profiles suitable for detecting diet variability within the region? and 4) Are there detectable changes in diet over time? To answer these questions, this study integrates contextual and osteobiographical data with stable isotope analysis (δ^13^C, δ^15^N) to evaluate dietary variation among Classic period populations in southeastern Petén. We compare these results with data from other major Central Lowland centers to examine pan-regional differences and further assess the potential influence of sex, age-at-death, status, and permanent body modifications on dietary intake in the relatively egalitarian social network that characterized southeastern Petén populations. Finally, we examine temporal shifts in their food consumption to gain a deeper understanding of the adaptative subsistence strategies employed in their mountainous and riverine homelands.

### Stable isotope systems

A range of archaeological methods, including protocols derived from paleobotany and zooarchaeology, have long been employed to gain insight into the food resources available to past human societies [8]. Increasingly, however stable isotope analysis has been employed because it provides a direct method for reconstructing a particular dietary intake offering insights into an individual’s diet across their lifetime [3], as the elemental and isotopic composition of human tissues reflects the average of foods consumed over time.

Carbon isotope ratios (δ^13^C) closely reflect primary producers (plants, algae, bacteria) at the base of food webs. They remain either relatively unchanged or undergo calculable changes (fractionation) through trophic transfers and biosynthesis in consumers and therefore closely resemble these base sources. Bone collagen δ^13^C (δ^13^C_coll_) values primarily represent the isotopic composition of dietary protein, which in omnivores is heavily weighted towards animal-based proteins but is also dependent on diet quality and individual nutritional state. By contrast, δ^13^C values from the carbonate group in hydroxyapatite of dental enamel (δ^13^C_enam_) represent the average carbon isotopic composition of the total diet (carbohydrates, lipids, and some protein, dependent on the exact composition of diet and energy metabolism pathways) during the period of enamel formation, typically *in utero* or during childhood [9,10]. δ^13^C values of plants are determined by the photosynthetic pathway they employ to fix CO_2_ from the atmosphere. Plants that use the C_4_ pathway, such as maize, incorporate more of the heavy isotope of carbon (^13^C) relative to the lighter isotope of carbon (^12^C) during photosynthesis compared to plants that use the C_3_ pathway. This leads to higher δ^13^C values in C_4_ plants (~ −12‰), when compared to C_3_ plants (~ −26 to −28‰), such as beans and squash [11,12]. These isotopic values are transferred to consumers with minimal enrichment (~1‰ for each trophic level) [13]. As maize (*Zea mays* spp.) is a C_4_ plant that significantly sustained ancient Mesoamerican populations [14], elevated δ^13^C_coll_ values in humans can reflect direct maize consumption or, still more likely, the consumption of animals raised on maize-based fodder. Meanwhile, elevated δ^13^C_enam_ values tend to reflect direct maize consumption as they are more closely tied to the plant portion of omnivorous diets. Consequently, analysis of δ^13^C_coll_ and δ^13^C_enam_ informs the relative contributions of C_3_ and C_4_ resources to past diets [13].

Nitrogen isotope ratios (δ^15^N) in bone collagen provide complementary information about trophic level and protein source. The δ^15^N values of consumers typically increase by 3–5‰ relative to their diet, reflecting stepwise enrichment with each trophic transfer. Individuals whose diets were based mainly on terrestrial herbivores exhibit lower δ^15^N values than those who consumed omnivorous or carnivorous species. Nursing infants have higher δ^15^N than their mothers, as consumption of breast milk effectively places them one trophic level above the maternal diet [15].

### Archaeological sites explored in this study

Ancient Maya society thrived across a vast and heterogeneous territory, encompassing plains, low hills, mountains, and metamorphic regions, where they developed unique subsistence strategies tailored to each area and its micro-environments. Some recent studies, such as those by López [6], have revealed that diets among populations in the Coastal area, Northern Maya Lowlands, and Southeast Periphery (Copan) remained consistent over time, especially during the Late and Terminal Classic periods. By contrast, changes in food consumption among Central Lowland populations have been documented in the same timeframe. Although early research indicates a similar diet across major Maya Lowland sites, such as Piedras Negras, Altar de Sacrificios, Seibal, Tikal, Pacbitun, Lamanai, and Uaxactun [16–21], little is known about the subsistence strategies of smaller cities, which are the focus of the present study. The Petén region, situated in northern Guatemala, boasts a diverse landscape, comprised of *bajos* with marshlands and lakes, rivers, open savannahs, limestone ridges, and high mountains [22]. Although publicly known for its monumental Maya centers, the Petén was also home to many smaller but nevertheless complex cityscapes in its southeastern fringes, including ruling centers and caves occupied from the Preclassic to the Early Postclassic periods. Since 1987, the *Atlas Arqueológico de Guatemala* Project, officially initiated by Juan Pedro Laporte, has systematically surveyed and mapped ceremonial and residential features of archaeological sites, along with their natural surroundings. From the start, its focus has not been the urban ruling elites but the commoner sociopolitical systems that characterize the western fringes of the Maya Mountains [22,23]. To date, more than 400 sites have been systematically documented, including information regarding topography, chronology, and population settlement patterns, among other relevant aspects [22]. However, the relationships between these smaller sites and their shared cultural and economic networks remain to be fully explored and understood, giving rise to the present research.

**Fig 1:**
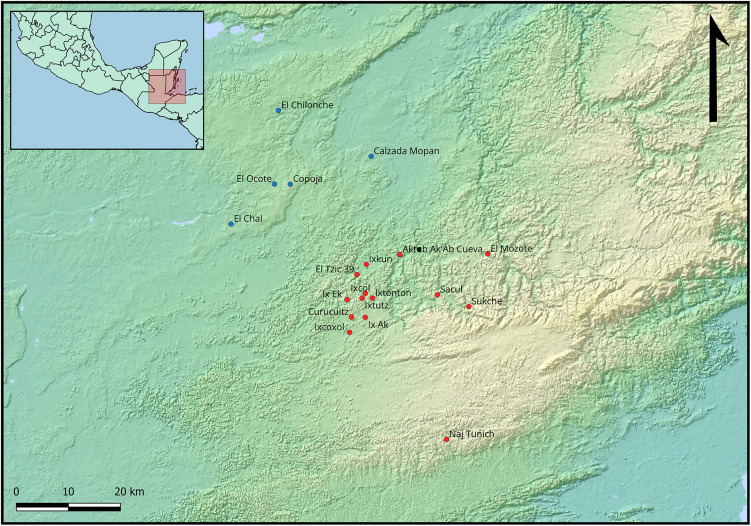
Map indicating the sites of southeastern Petén, Guatemala discussed in the study according the geographic subregions: Maya Mountains (red dots) and humid savannah (blue dots). LiDAR base map by Dr. Charles Golden (ArcGIS) and modified by Karla Arjona published under Creative Commons Attribution License (CC-BY 4.0).

The sample selected for this study dates to the Classic period. Site populations are divided into two clearly defined areas of southeastern Petén, characterized by their orographic features: these correspond first to the western part of the Maya Mountains, and second, to the northern humid savanna (Fig 1). The former is located on the Dolores-Poptun plateau, where tropical forests overlook the Mopan River plains. This area features settlements such as Ixtutz and its smaller centers, including Ixcoxol, located in the Poxté River basin. Ixtonton is perhaps the largest center in this area, both in size and economy, while Ix Ak is counted among its noted secondary centers. A second primary center, Ixkun, includes El Tzic and the Aktun Ak’ab cave as secondary sites. Ixcol, with its minor site Sukche, also occupies this plateau. These settlements have been interpreted as the food basket of the Petén: communities with intensive agricultural productivity coupled with high residential density. This differed from the economic orientation of nearby Curucuitz, specialized in exploiting pine forest resources [24]. Additional orographic sites, like the well-known Naj Tunich cave, were used by the Maya for ritual purposes. Still higher in elevation are the settlements of Sacul, located in the basin of the eponymous river, and Ix Ek, in the Yaltutu mountain range, a steeper terrain home still today to complex residential clusters with active occupation. Finally, El Mozote, situated on a broad hilltop along the border between Belize and Guatemala, is located in the upper basin of the Chiquibul River [7].

Subsistence strategies varied distinctly between the Maya Mountains and the adjacent northern humid savanna zone, characterized by extensive floodplains in the middle basin of the Mopan River and the upper basin of the San Juan River. These hydrological systems fertilize the surrounding soils, making them highly suitable for cultivation. Here, most settlements were established on more elevated terrain. Calzada Mopan is a prominent site within the middle Mopan river valley notable for its high population density, diverse residential architecture (ranging from simple to complex units), and the presence of four ballcourts [25,26]. In the San Juan Basin, Copojá and El Ocote sites are distinguished by continuous occupation beginning in the Late Preclassic period [26]. El Chal, located amid well-drained uplands, represents one of the largest centers in southeastern Petén. Towards the fringes of the modern-day town of Dolores lies El Chilonché, a large center marked by monumental architecture, including tall platforms and broad plazas.

Most of these entities described in southeastern Petén, whether higher-ranking or secondary sites, share typical elements of Classic Maya urbanism, including E-Groups, acropolis complexes, ballcourts, and causeways (*calzadas*). Such characteristics suggest that these communities were politically and economically stable, comparable to the major centers of the northern Petén without sharing their degree of centralization [7].

## Materials and methods

### Human remains

The human skeletal materials analyzed for southeastern Petén were recovered mainly from non-elite residential and burial contexts. The chronology of the burials has been determined primarily through the typology of associated ceramics. For burials without associated ceramic wares, the stratigraphy or special features as presence of stuccoed floors, filling features, immediate architectural association, and analysis of burial patterns were examined. The wealth of each burial was estimated by scoring a set of attributes. A crude score was calculated for each burial using a standard set of attributes present. Thus, six classes of burial contexts—“0” (lowest, no offering), to “5” (highest, more than six status indicators)—were distinguished according to the presence of a choice of 17 different status markers in all primary individual interments, a list adjusted and extended from Krejci and Culbert [27] by Tiesler [28].

Sampling was conducted after obtaining permits issued by the Guatemalan Institute of Anthropology and History (IDAEH) to the Atlas Project, currently coordinated by Lilian Corzo and Mara Reyes (Tables 1 and 2). Subsequently, collagen was extracted from a total of 57 bone samples for δ^13^C and δ^15^N and enamel was obtained from 37 permanent teeth to analyze the carbonate group in bioapatite. Teeth were chosen for their completeness, absence of visible pathology, and well-preserved enamel. When multiple teeth were present, first molars were prioritized to provide information on early childhood diet, as their crowns form and mineralize between approximately one and five years of age. Long bones (primarily femora and humeri) were preferentially selected when available to ensure comparable growth timeframes.

**Table 1:** Collagen stable isotope data from southeastern Petén bone samples.

Site	Burial	Bone segment	Burial type	Chronology^a^	Sex	Age^b^	Dental decoration	Cranial modification	Status^c^	δ^15^N	δ^13^C	C:N
**Ak’ab Cave**	170	Left petrous	Cave	TC	F	Adult	^d^	Tabular oblique mimetic	1	9	−9	2.7
**Chilonche**	189	Left femur	Pit	TC	–	Adolescent	NO	Tabular oblique mimetic	1	8.5	−8.6	2.7
**Copoja**	137C	Right petrous	Limestone-carved pit	LP	–	1 INF	^d^	^d^	2	11.4	−17.2	3.4
**Curucuitz**	126	Femur NL	Cist	LC	F	Adult	NO	^d^	0	7.2	−10.7	3
**Curucuitz**	149	Left petrous	Pit, Natural rock	TC	–	1 INF	^d^	^d^	0	9.9	−10.7	2.8
**Curucuitz**	150	Right petrous	Limestone carved pit	TC	F	Adult	NO	^d^	1	7.6	−11.1	2.7
**Curucuitz**	160	Femur NL	Cist	LC	F	Adolescent	^d^	^d^	1	7.4	−11	2.8
**Curucuitz**	185A	Tibia NL	Chultun	EC	–	Adult	^d^	^d^	1	8.2	−11.5	3.1
**El Chal**	068A	Right femur	Pit/cover with limestone	LP	–	Adolescent	^d^	^d^	1	8.6	−10.5	2.9
**El Chal**	272A	Right femur	Cist	TC	–	Adolescent	^d^	YES	1	9.1	−11.2	2.7
**El Chal**	272B	Left femur	Cist	TC	–	Adult	^d^	^d^	1	10.4	−8.7	2.8
**El Mozote**	197	Right petrous	Limestone carved pit	LC	M	Adolescent	^d^	NO	1	9.2	−8.5	2.8
**El Ocote**	136B	Left petrous	Simple	LC	–	2 INF	NO	^d^	0	8.8	−15.2	3
**El Tzic 39**	48	Left tibia	Cist	LC	–	Adult	NO	^d^	1	7.9	−7.9	2.7
**Ix Ak**	35	Long bone	–	LP	M	Adolescent	^d^	^d^	1	7.8	−8.8	2.8
**Ix Ek**	83	Femur NL	–	LC	M	Adolescent	^d^	^d^	0	7.9	−10.2	2.8
**Ix Ek**	84	Femur NL	–	LC	–	Adolescent	^d^	^d^	0	8.5	−9.4	2.8
**Ix Ek**	90	Long bone	–	LC	–	Adolescent	^d^	^d^	0	–	–	
**Ix Ek**	110A	Femur NL	Cist	LC	–	SADO	NO	NO	1	8.4	−9.5	2.9
**Ix Ek**	112	Femur NL	Cist	LC	M	SADO	Filing B4	Tabular erect	0	8.4	−9	2.7
**Ixcoxol**	135	Left tibia	Cist	–	M	Adult	Inlay E1	Tabular oblique mimetic	1	8.9	−9.2	2.8
**Ixkun**	50	Right femur	Simple	LC	M	Adult	^d^	^d^	1	9.2	−8.8	2.9
**Ixkun**	57	Femur NL	Pit	EC	–	Adolescent	^d^	^d^	0	8.5	−9	2.9
**Ixkun**	168	Tibia NL	Cist	TC	–	SADO	^d^	^d^	1	9.2	−8.8	2.8
**Ixkun**	258	Right femur	Cist	LC	–	Adolescent	^d^	^d^	2	8.4	−9.3	2.8
**Ixtonton**	4	Left femur	–	EC	–	d	^d^	^d^	1	7.8	−10.3	2.9
**Ixtonton**	6	Right femur	Cist	LC	M	Adult	^d^	NO	1	8.3	−10.8	3
**Ixtonton**	28	Femur NL	Simple	TC	M	SADO	NO	Tabular	0	9.4	−8.7	2.8
**Ixtonton**	31	Femur NL	Simple	TC	F	SADO	Filing B4 A2	NO	1	9.1	−10.1	2.9
**Ixtonton**	32	Tibia NL	Simple	TC	F	SADO	^d^	^d^	1	9	−10.3	2.8
**Ixtonton**	54	Femur NL	Cist	LC	–	Adolescent	^d^	^d^	0	9.2	−11.8	2.7
**Ixtonton**	55	Femur NL	Chamber	TC	–	Adolescent	^d^	^d^	2	7.7	−8.7	2.8
**Ixtonton**	62	Left femur	Cist	TC	M	Adult	Inlay E1 Filing A4	NO	1	8.8	−10.9	3.2
**Ixtonton**	92	Femur NL	Cist	TC	–	SADO	NO	NO	1	10.1	−8.8	2.7
**Ixtonton**	93	Femur NL	Cist	LC	–	SADO	^d^	^d^	1	8.6	−10.2	2.8
**Ixtonton**	94	Femur NL	Cist	LC	–	Adolescent	^d^	^d^	1	8.8	−8.7	2.8
**Ixtonton**	102	Right fibula	Cist	TC	–	Adolescent	^d^	^d^	1	8.6	−8.5	2.8
**Ixtonton**	104	Left femur	Cist	LC	–	^d^	^d^	^d^	1	8.7	−8.4	2.7
**Ixtonton**	106	Right fibula	Cist	TC	F	SADO	Filing B4 A2	Tabular oblique extreme	1	8.2	−9.1	2.8
**Ixtonton**	143	Femur NL	–	LC	M	Adolescent	^d^	^d^	0	8.1	−8.6	2.8
**Ixtonton**	235	Femur NL	Cist	LC	–	Adolescent	^d^	^d^	0	8.1	−8.8	2.8
**Ixtutz**	172	Tibia NL	–	LC	F	Adult	^d^	^d^	1	7.8	−10.4	2.8
**Ixtutz**	173	Left tibia	–	LC	M	SADO	^d^	^d^	1	8.2	−8.2	2.8
**Ixtutz**	322	Right tibia	–	LC	–	1 INF	^d^	^d^	^d^	9	−8.8	2.8
**Ixtutz**	324 FEM2	Right femur	Pit	LP	–	Adolescent	NO	^d^	^d^	8.5	−9.6	2.8
**Ixtutz**	324 FEM7	Left femur	Pit	LP	–	Adolescent	NO	^d^	^d^	8.3	−10.9	2.8
**Sacul**	175B	Left femur	Cist	TC	M	Adolescent	^d^	YES	3	8.7	−8.7	2.7
**Sukche**	74	Left tibia	–	LC	–	Adolescent	^d^	^d^	0	9.9	−9.8	2.7

NL: Non-lateralized.

^a^LP: Late Preclassic, EC: Early Classic, LC: Late Classic, TC: Terminal Classic.

^b^1 INF: 0–2.5 years, 2 INF: 2.5–5.5 years, Adolescent: 10–15 years, SADO: 15–25 years, Adult: 25–55 years, according to Tiesler [34].

^c^According to Krejci and Culbert [27].

^d^Not determined.

**Table 2:** Carbonate enamel stable isotope data.

Site	Burial	Tooth^a^	Burial type	Chronology^b^	Sex	Age^c^	Dental decoration	Cranial modification	Status^d^	δ^13^C
**Calz. Mopan**	212A	46	Cist	TC	–	INF 3	^e^	YES	^e^	−4.8
**Calz. Mopan**	212B	36	Simple	TC	–	INF 1	^e^	YES	^e^	−10.2
**Calz. Mopan**	212D	16	Simple	TC	–	INF 3	^e^	NO	^e^	−9.1
**Calz. Mopan**	212E	36	Simple	TC	–	INF 1	^e^	Tabular oblique	^e^	−12.1
**Calz. Mopan**	212H	26	Simple	TC	M	Adult	^e^	NO	^e^	−8.4
**Calz. Mopan**	213	36	Simple	EC	F	Adult	Filing C2	Tabular oblique	1	−3.3
**Calz. Mopan**	216	46	Pit	TC	M	Adult	NO	NO	1	−4.8
**Calz. Mopan**	220A	46	Pit	TC	F	Adult	Filing Ik	^e^	1	−4.1
**Calz. Mopan**	221C	26	Simple	TC	M	Adult	Inlay E	^e^	^e^	−2.2
**Calz. Mopan**	223	46	Pit	EC	–	INF 1	^e^	^e^	0	−3.9
**Calz. Mopan**	212F	37	Simple	TC	–	^e^	^e^	NO	^e^	−10.4
**Chilonche**	189	36	Pit	TC	–	Juvenile	NO	Tabular oblique	0	−2.5
**Cueva Ixkun**	312A, Ind. 17A	46	Simple	–	–	Adult	NO	NO	^e^	−3.5
**Cueva Ixkun**	245	16	Simple	LC	–	Adult	^e^	NO	^e^	−1.6
**Curucuitz**	126	46	Cist	LC	–	Adult	NO	^e^	0	−2.3
**Curucuitz**	150	26	Limestone carved pit	TC	F	Adult	NO	^e^	1	−4.5
**Curucuitz**	158	16	Limestone carved pit	–	M	Adult	Inlay E	^e^	1	−1.6
**El Chal**	264	15	Cist	LC	F	Adult	^e^	^e^	^e^	−7.7
**Ix Ek**	90	46	–	LC	–	Adult	^e^	^e^	0	−3.2
**Ix Ek**	112	36	Cist	LC	M	Adult	Filing B4	Tabular erect	1	−1.7
**Ixcol**	45	46	Cist	TC	–	Adult	Filing B4	^e^	0	−3.5
**Ixcoxol**	130	36	Cist	LC	–	Adult	Filing B4 C3 Inlay G2	^e^	1	−3.6
**Ixtonton**	6	17	Cist	LC	M	Adult	^e^	^e^	1	−5.2
**Ixtonton**	23A	46	Cist	TC	–	^e^	^e^	^e^	3	−1
**Ixtonton**	28	46	Pit	TC	M	Adult	NO	Tabular undetermined	0	−1.2
**Ixtonton**	31	46	Pit	TC	F	Adult	Filing B4 A1	^e^	1	−2.7
**Ixtonton**	51	26	Cist	LC	F	Adult	Filing B2 Inlay G3 G1	^e^	1	−2.4
**Ixtonton**	62	26	Cist	TC	M	Adult	Filing A1 Inlay G1	^e^	1	−5.9
**Ixtonton**	296	13	Cist	TC	M	Adult	^e^	^e^	1	−2.3
**Ixtonton**	010b?	17	Chamber	LC	–	Adult	^e^	^e^	4	−0.7
**Ixtutz**	324 Mand 2	26	Chamber	EC	–	Adult	NO	^e^	^e^	−4.1
**Ixtutz**	324, Mand 1	18	Chamber	EC	–	Adult	Sgraffito D7	Tabular erect?	^e^	−2.7
**Naj Tunich**	328	46	Simple	EC		^e^	^e^	^e^	^e^	−2.4
**Sacul**	176a	36	Cist	TC	–	Adult	NO	^e^	3	−2.9
**Sacul**	178	48	Cist	LC	F	Adult	Filing Ik B5	Tabular oblique	1	−2.2
**Sacul**	193	36	Cist	LC	M	Adult	Inlay E	^e^	1	−4.1
**Sacul**	194	46	Cist	LC	M	Adult	Inlay E	^e^	1	−2.4

^a^FDI dental nomenclature

^b^EC: Early Classic, LC: Late Classic, TC: Terminal Classic.

^c^1 INF: 0–2.5 years, 2 INF: 2.5–5.5 years, Juvenile: 15–25 years, Adult: 25–55 years, according to Tiesler [34].

^d^According to Krejci and Culbert [27].

^e^Not determined.

For this study, sex determination was conducted according to the most marked dimorphic morphological features: those of the pelvic girdle and the skull as described by Buikstra and Ubelaker [29]. This scrutiny was complemented, when applicable, with dimorphic discriminative standards developed by Wrobel et al. [30] and Tiesler [28]. For age-at-death estimation, dental and epiphyseal maturation criteria were used together with the degree of suture closure and the morphology of the pelvic pubic symphysis and auricular surfaces [29,31]. For the purposes of this research, we distinguished “child” ages-at-death (all under five years in our cohort) from juveniles (10–25 years of age-at-death) and adults (above 25 years of age-at-death). Permanent body modifications of a cultural nature, namely cranial vault modifications and dental works, were scored according to the typologies laid out by Romero [32] and Tiesler [33,34]. The former were categorized by type, variant, degree, and secondary shifts in cranial morphology, while the latter were evaluated in terms of their presence, technique, and filing contour designs.

### Bone collagen δ^13^C and δ^15^N analysis

Exterior bone surfaces were mechanically cleaned to remove adhered sediment and potential contaminants before sampling. Approximately 1.5–3.0 g of cortical bone from long bone shafts were processed for collagen extraction using a modified Longin protocol [35] with ultrafiltration [36,37]. Samples were demineralized in ~20 mL of 0.5 N HCl at 5°C for 24–72 hours, until complete mineral dissolution was achieved, followed by three rinses with 18.2 megohm/cm H_2_O to achieve neutrality. The resulting collagen pseudomorphs were freeze-dried and weighed to assess preservation. Collagen was gelatinized in 20 mL 0.01 N HCl at 67°C for 12 hours, and the supernatant was pipetted into sterile 50 mL tubes and lyophilized for 48–72 hours. The process was repeated to maximize collagen yield, and the resulting gels were combined and resuspended in ~2 mL of ultrapure water. The solution was filtered through pre-cleaned Sartorius Vivaspin®20, 30 kDa MWCO Polyethersulfone ultrafilters and centrifuged three times [29], reducing the volume to ~0.5 mL to 1.5 mL. The purified collagen was then lyophilized for 48 hours and weighed to determine the final yield.

For δ^13^C and δ^15^N analysis, ~ 0.7 mg of purified collagen was weighed into 3 × 5 mm tin capsules and analyzed using a Costech 4010 Elemental Analyzer (Valencia, CA) connected to a Thermo Scientific Delta V Plus isotope ratio mass spectrometer (Bremen, Germany) at the University of New Mexico Center for Stable Isotopes (UNM-CSI). Stable isotope data are reported in δ notation per mil (‰) using the equation δ^13^C or δ^15^N = 1,000*[(R_sample_/R_standard) –_ 1], where the R_sample_ and R_standard_ represent the ratios ^13^C:^12^C or ^15^N:^14^N in the sample and standard. Analytical precision was based on replicate analysis of protein-based internal reference material. All standard deviations were less than 0.2‰ for δ^13^C and δ^15^N values. δ values are expressed relative to internationally accepted standards: Atmospheric N_2_ for δ^15^N and Vienna-Pee Dee Belemnite for δ^13^C. Collagen quality was evaluated according to established criteria: atomic C/N ratios between 2.9 and 3.6, collagen yield greater than 1%, and %C and %N values within acceptable ranges for well-preserved archaeological collagen (Table 1) [30,31].

### Enamel δ^13^C analysis

Enamel surfaces were mechanically cleaned using a rotary tool fitted with a precleaned 200 µm carbide endmill or diamond burrs to remove adhered material and expose clean enamel (S2 Table, S1 File). Approximately ~15–20 mg of enamel powder was collected for isotopic analysis. The structural integrity of enamel carbonate was assessed using Fourier Transform Infrared Spectroscopy (FTIR) to ensure minimal diagenetic alteration. Enamel chips were crushed with an agate mortar and pestle. The resulting powder was transferred to precleaned 2 mL centrifuge tubes and treated with 0.1 M acetic acid (0.1 mL per mg of sample) to remove secondary carbonates. The samples were gently agitated for ~30 seconds and allowed to react for no more than 4 hours. They were then rinsed to neutrality with 18.2 megohm/cm H_2_O and centrifuged for 1 minute at 3000 rpm between each rinse. After the final rinse, the samples were freeze-dried overnight (~12 hours). For isotopic analysis, ~ 5 mg of cleaned, dried enamel powder was weighed into 12 mL glass exetainers and analyzed on a Gasbench device coupled to a Thermo Fisher Scientific Delta XL Advantage for δ^13^C and δ^18^O from the carbonate fraction of bone bioapatite. Isotopic results are reported in per mil (‰) relative to the VPDB standard. Analytical precision, based on replicate analyses of carbonate standards (NBS-19, NBS-18, and internal calcite), was better than ± 0.1 ‰ for δ¹³C and ± 0.2 ‰ for δ¹⁸O. To determine the degree of apatite recrystallization, the crystallinity index or infrared splitting factor was calculated using the height of the absorption bands at 603 and 565 cm^−1^ divided by the height of the valley between them at ~595 cm^−1^, while the ratio of the absorption peak height at 1415 cm^−1^ (CO_3_) and 1035 cm^−1^ (PO_4_) (C/P) was calculated to evaluate the samples for degraded carbonate material. All sample spectra had a crystallinity index less than 3.8 and C/P ratios >0.125 which were consistent with those expected in well-preserved archeological bone [38].

### Statistical analysis

To evaluate local foodways in the southeastern Petén region, the isotopic data obtained in this study were processed collectively, then compared with published δ^13^C and δ^15^N datasets from other Maya Lowland areas. Comparative data were compiled into a database maintained by the Bioarchaeology Laboratory of the *Universidad Autónoma de Yucatán*, which also included profiling contextual and osteobiographical information for each individual.

Statistical analyses were conducted using the R 4.4.1 environment. All tests were two-tailed with a significance threshold of *p* > 0.05. Individual burials were categorized by isotopic composition and contextual variables, including region, associated residential complex, age-at-death, sex, burial mode and type, inferred social status, presence of dental decoration or cranial modification, and chronological period. Normality of the data was assessed using the Lilliefors test (a modified Kolmogorov-Smirnov test; nortest package v. 1.0.4 [39]), and homogeneity of variance was evaluated using Bartlett’s test (stats package v. 4.4.1 [40]). When the assumption of heterogeneity was violated, a nonparametric Kruskal-Wallis test was applied, followed by Bonferroni-corrected post hoc pairwise comparisons. Thus, Kruskal-Wallis tests were performed on isotopic data according region, residential complex, age-at-death, sex, burial mode, burial type, status, dental decoration, and cranial modification from collagen and enamel samples, while ANOVA tests were carried out on collagen and enamel isotope values according to chronology. Descriptive and comparative plots were produced in R using the ggplot2 package v. 3.5.1 [41] (tidyverse framework). These tests were chosen to accommodate variable sample sizes, potential deviations from normality, and heterogeneity of variance among isotopic groups.

## Results

Tables 1 and 2 summarize the stable isotope results, contextual information, and collagen/enamel quality parameters for the individuals from southeastern Petén. S1 Table (S1 File) present the summary and comparative statistics contrasting individuals from southeastern Petén with published isotope values from other Classic Maya sites across the Central Lowlands (CLL). Summary statistics are grouped by their δ^13^C_coll_ values, residential complex (Ixtonton), age-at-death, sex, burial mode and type, inferred social status, dental decoration, cranial modification, and chronological subperiods (S2–S6 Tables, S1 File). All 57 collagen samples passed quality control, with collagen yields exceeding 1% and atomic C/N ratios ranging from 2.9 to 3.6 [42]. Likewise, the 37 enamel samples produced acceptable C/P ratios and crystallinity indexes determined via FTIR analysis. Non-parametric Kruskal-Wallis tests with Bonferroni-adjusted post hoc comparisons were applied to data groups with non-normal distributions (S7 Table, S1 File).

### Regional comparisons

**Fig 2:**
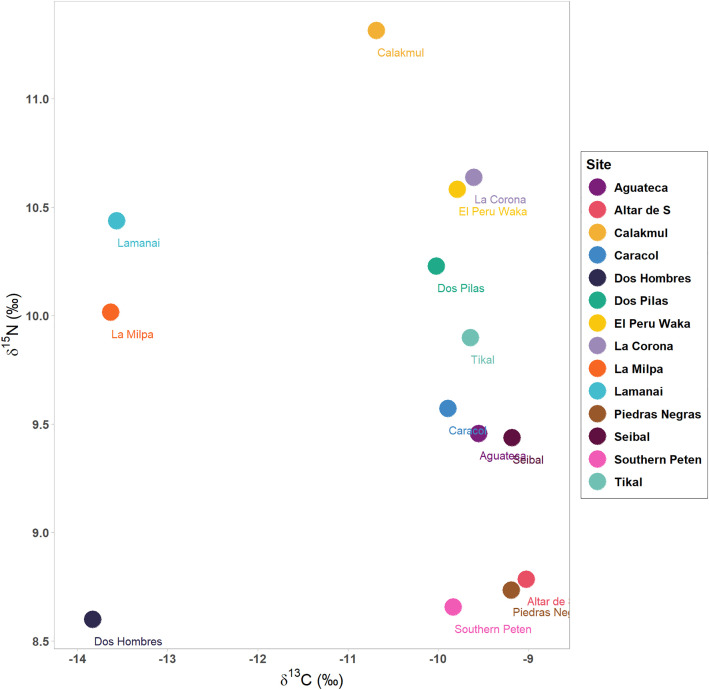
Scatterplot of mean δ^13^C and δ^15^N collagen values for southeastern Petén compared to other sites of the Central and Southeastern Lowlands.

Mean δ^13^C_coll_ and δ^15^N_coll_ values for the southeastern Petén sites are provided in S1 Table (S1 File). Fig 2 situates the average δ^13^C and δ^15^N collagen values for southeastern Petén and contextualizes them with those from other Classic-period sites representing major subregions of the Maya Lowlands as defined by Chase et al. [43]: Calakmul (zone 3) [44], Tikal (zone 4) [19], La Milpa, Dos Hombres and Lamanai (zone 5) [21,45], Caracol (zone 6) [46], Dos Pilas, Seibal, Aguateca and Altar de Sacrificios (zone 8) [17]. Three additional sites along the Rio Candelaria, Rio San Pedro, and Rio Usumacinta – La Corona, El Peru Waka’, and Piedras Negras – were also included in this regional comparison [16,47] (LBA/UADY Database).

**Fig 3:**
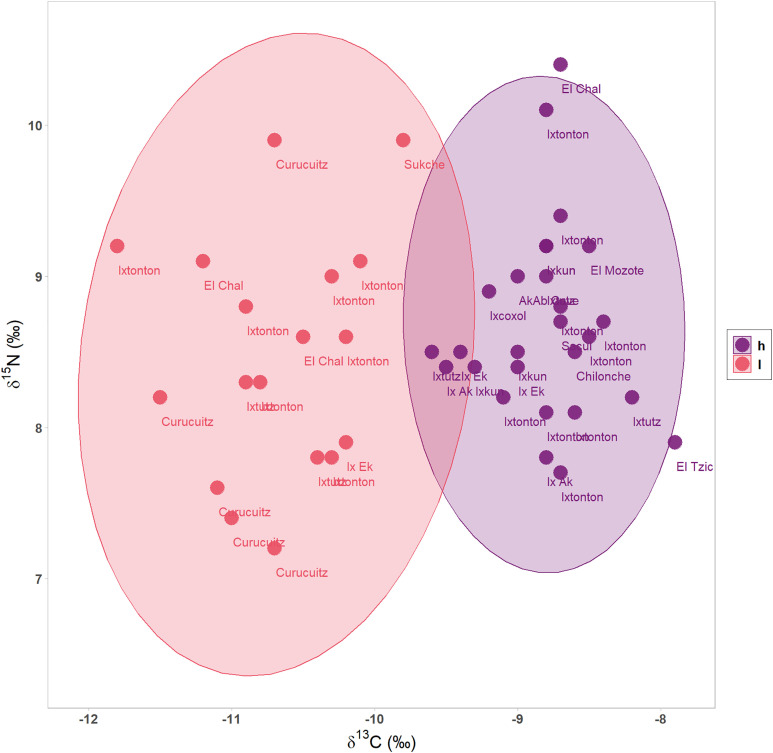
Scatterplot of δ^13^C and δ^15^N collagen values for southeastern Petén sites showing ellipses according their distinctive δ^13^C levels. *h:* Individuals with a diet highly dependent on maize and animals fed with this crop, according to its enriched values. *l:* Individuals with less negative carbon values. Ellipses represent a 95% confidence level for multivariate *t*-distribution.

Kruskal-Wallis tests revealed no significant isotopic differences between southeastern Petén and most comparative regions, except for zone 5 in Belize, which exhibited significantly lower (more negative) δ^13^C_coll_ values (*p* < 0.05; S1 Table, S1 File). Significant pairwise differences were observed between southeastern Petén and the sites of Dos Hombres, La Corona, Lamanai, and Seibal for δ^13^C_coll_ values, and Calakmul, Caracol, and Lamanai for δ^15^N_coll_ values (S1 Table, S1 File; Fig 2). Individuals with high and low δ^13^C_coll_ values identified from the 95% confidence ellipses in bivariate scatterplots also differed significantly (*p* < 0.05; S2 Table, S1 File; Fig 3). No significant differences were observed among individuals from distinctive residential complexes from Ixtonton site (*p* > 0.1) or between those from sites in the Maya Mountains and the humid savannah region covered by the Atlas Project (*p* = 0.1056; S2 Table, S1 File).

### Intra-population and demographic variation

Kruskal-Wallis tests according to age-at-death, sex, burial mode, burial type, status, dental decoration, and cranial modification on collagen and enamel samples revealed that food practices were broadly similar across sex, burial type (primary simple deposition, pits, cists, limestone pits, and funerary chambers), and social or identity markers such as dental decoration and cranial modification (S7 Table, S1 File).

Conversely, δ^13^C_coll_ and δ^13^C_enam_ values differed significantly between “Child” and “Juvenile” categories (S3 and S7 Tables, S1 File). While collagen could suggest changes in protein composition during growth and development stages, the differences in δ^15^N_coll_ values which approached statistical significance (*p* = 0.0729) probably owe to a weaning signal (most of them were categorized as 0–2.5 years). Meanwhile, enamel values from the “Child” category (*p* = 0.0136) were not representative enough to assert this change in southeastern Petén, since they correspond to the same burial.

In addition, δ^13^C_enam_ values differed significantly between individuals from primary and secondary burials (*p* = 0.0483) (S4 and S7 Tables, S1 File), suggesting some variation in diet between individuals in these mortuary contexts. Those with cranial modification had higher and more consistent δ^13^C_enam_ values, indicating a greater reliance on C4-based resources, such as maize (S5 and S7 Tables, S1 File).

Furthermore, ANOVA tests of collagen and enamel δ^13^C and δ^15^N_coll_ values according to chronology revealed no substantial temporal shifts in macronutrient consumption (S6 and S7 Tables, S1 File), although δ^15^N_coll_ values show a marginal increase towards the Terminal Classic, relative to the Late Classic (*p* = 0.2680).

## Discussion

### Dietary composition and maize dependence

Isotopic data from southeastern Petén indicate that its Classic-period populations relied heavily on C_4_-based resources, while also incorporating different proportions of C_3_ plants and C_3_-consuming terrestrial fauna into their diets. The average δ^13^C_coll_ values indicate a substantial contribution of C_4_-derived protein, translating to intensive consumption of maize-fed animals and/or direct intake of maize and maize products. The δ^13^C_enam_ values, which integrate carbon from all macronutrients (in proportions dependent on diet composition) eaten during enamel formation, equally reflect high C_4_ consumption, confirming that maize not only served as a staple food and fodder grain, but was a major caloric source. The overlap of δ^13^C_coll_ and δ^13^C_enam_ values across sites suggests that dietary patterns emphasizing maize persisted through different life stages. Elevated δ^13^C_enam_ values in individuals with cranial modification, which is an identity marker in many Classic communities, may indicate that differences in access to or preference for maize-based foods were socially mediated. The isotopic enrichment observed in these individuals, although modest, supports the interpretation that maize consumption was both nutritionally and symbolically central to Classic commoner Lowland Maya people.

### Southeastern Petén at a regional level

Comparative analysis of δ^13^C_coll_ values with other Central Lowland regions shows that southeastern Petén populations were broadly similar to those of contemporaneous sites such as Tikal, Calakmul, and Caracol, further reinforcing the idea that maize dependence was widespread across social and geographic contexts. The significantly depleted δ^13^C_coll_ values observed in some of the Belizean sites (zone 5, by contrast) likely reflect a higher consumption of domestic and/or wild C_3_ resources such as root vegetables, legumes, palms, and, importantly, forest fauna, rather than fundamental differences in organized subsistence infrastructures. Overall, the isotopic evidence places Petén’s southeastern communities within the broader pattern of Classic Maya agricultural dependence and highlight local flexibility in dietary composition that was likely both socially and ecologically mediated.

The isotopic similarity between southeastern Petén and most Central Lowland populations reflects the proportions of C_4_-based protein intake (reflected in δ^13^C_coll_) and underscores the adaptive ingenuity of Maya agricultural systems. Across the Maya Lowlands, communities developed localized strategies to ensure food security in distinct ecological zones, such as modifying karst plateaus in Calakmul and Caracol, managing wetlands near Blue Creek, and modifying upland forests around Tikal through slope terracing, box terraces, and check dams [48]. Pertaining to southeastern Petén, the alluvial soils in the Dolores Valley remain fertile today and were historically exploited through shifting (slash-and-burn) cultivation. Agricultural terraces constructed in the steep limestone hills surrounding many Atlas sites further supported intensive production [49], contributing to the stability of long-term subsistence that allowed several centers to remain occupied well into the Postclassic period [49,50].

The lower δ^13^C_coll_ values observed for sites in the Blue Creek region (such as La Milpa, Dos Hombres, and Lamanai) likely reflect differences in land-use histories rather than dietary preferences. These areas experienced early and persistent soil exploitation beginning in the Preclassic period, with agricultural intensification through field walls and terracing continuing into the ninth century CE. After this period, soil exhaustion may have necessitated an increased reliance on market-based food redistribution [51,52].

Southeastern Petén’s comparatively low δ^15^N_coll_ values place it among other large central sites to its west, such as Piedras Negras and Altar de Sacrificios, both in the Río de la Pasión Basin (Table 1). Both site populations seem to have infrequent consumption of aquatic resources in common with the Atlas regional series, when compared with further Lowland Maya populations. This pattern may have resulted from the reduced availability of fish species in local waterways or limited use of fishing technologies, as has been proposed for Piedras Negras [16]. The combination of high δ^13^C values (in collagen and enamel) and relatively low δ^15^N_coll_ values indicates a heavy dependence on maize and other land-based foods, as opposed to aquatic resources, emphasizing the region’s focus on terrestrial subsistence strategies within a diverse ecological landscape.

### Foodways among classic-period southeastern Petén populations

While maize formed the foundation of Classic-period subsistence across the Central Lowlands, populations in southeastern Petén employed diverse strategies to supplement this staple with locally available plant and animal resources. The isotopic variation among individuals from this region points to meaningful dietary differentiation at both inter- and intra-site scales. Two primary clusters are evident in the δ^13^C and δ^15^N spaces of collagen (Fig 3). Individuals in the first cluster show higher δ^13^C_coll_ values, indicating a greater dependence on maize and/or animals raised or feeding in maize fields, such as deer, peccaries, dogs, and turkeys [53,54]. The second cluster, characterized by more negative δ^13^C_coll_ values, suggests a mixed diet, incorporating more C_3_ plants and potentially lower-trophic terrestrial fauna. These patterns are consistent with differing local ecologies and land-use histories. A Kruskal-Wallis test confirmed the significant differences between the two groups (*p* = 0.000017), with more negative δ^13^C_coll_ values (−11.8 to −9.8‰) associated with urban individuals from Ixtonton, El Chal, and Curucuitz, and the less negative δ^13^C_coll_ values (−9.6 to −7.9‰) found mainly among populations in the Mopan basin and the humid savanna (Fig 3).

**Fig 4:**
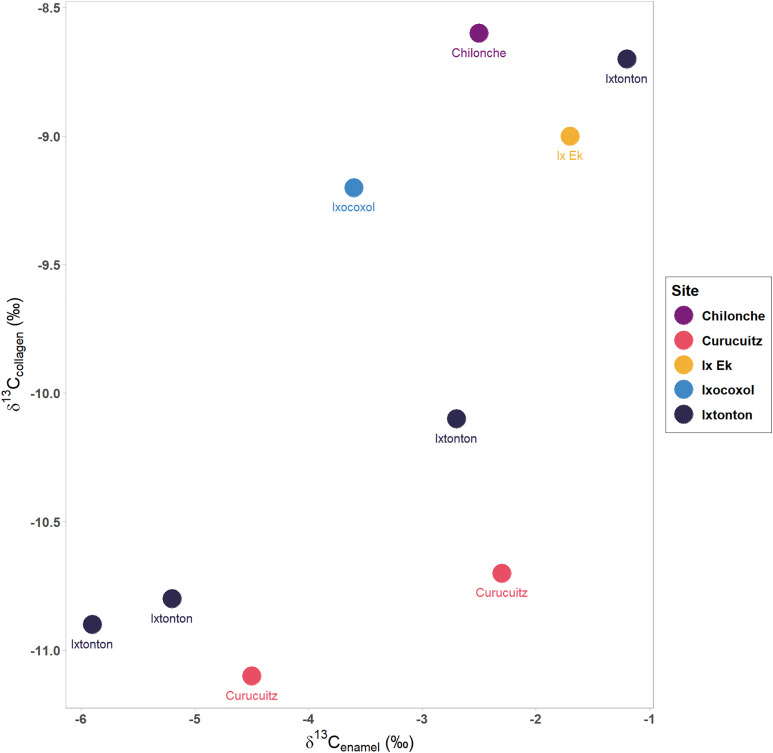
Scatterplot of δ^13^C_enam_ vs. δ^13^C_coll_ values from paired samples of southeastern Petén.

The distinction between collagen and enamel values further substantiates these dietary contrasts. When δ^13^C_coll_ is confronted with δ^13^C_enam_ (Fig 4), individuals from Curucuitz and some from Ixtonton fall below the C_4_/marine protein line, consistent with their diets having been enriched with C_3_ resources. By contrast, four individuals from Ix Ek, Ixtonton, Ixcoxol and El Chilonché present less negative δ^13^C values in both tissues, suggesting more substantial reliance on maize-based foods and maize-eating animals.

Site-level differences align well with further archaeological and environmental trends. Curucuitz, a smaller center contemporaneous with Ixtonton, flourished during the Late Preclassic and again during the Late Classic, despite occupying marginal terrain with poor soils, erosion, and drainage issues [24,55]. These conditions likely limited agricultural activities and encouraged a greater use of forest and C_3_-based resources, which is reflected in the lower δ^13^C_coll_ and δ^15^N (> 8.5‰) values documented in this paper. Conversely, individuals from El Chal’s sampled population exhibit higher δ^15^N_coll_ values, suggesting a greater consumption of higher-trophic fauna, such as terrestrial omnivores/carnivores or aquatic species from the nearby lagoons of Oquevix or Ijá [56]. The relatively low δ^15^N values across much of the Petén region, including among El Chal’s burial population, are consistent with the general absence of fish remains in local archaeofaunal assemblages [57].

**Fig 5:**
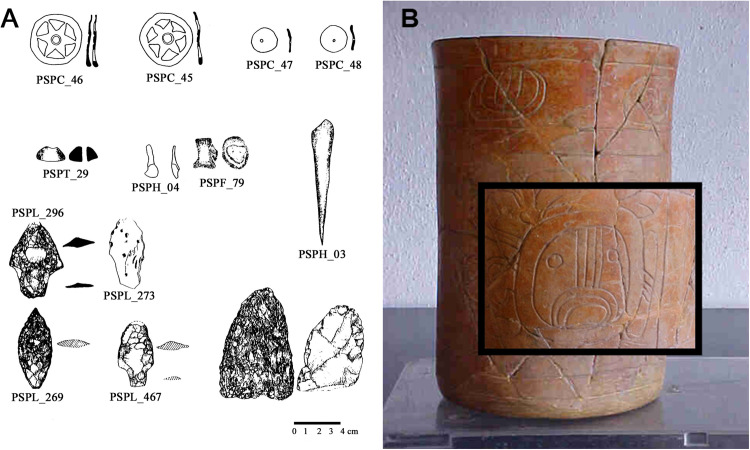
A. Ornamental and utilitarian artifacts recovered during explorations of the C Residential Complex. **B.** Cylindrical incised Torres-type vessel: Torres (A-502) found as an offering in Burial 94, near Central Ixtonton. Images with institutional copyright by the Archaeological Atlas of Guatemala project coordinated by Mara Reyes and published under Creative Commons Attribution License (CC-BY 4.0).

**Fig 6:**
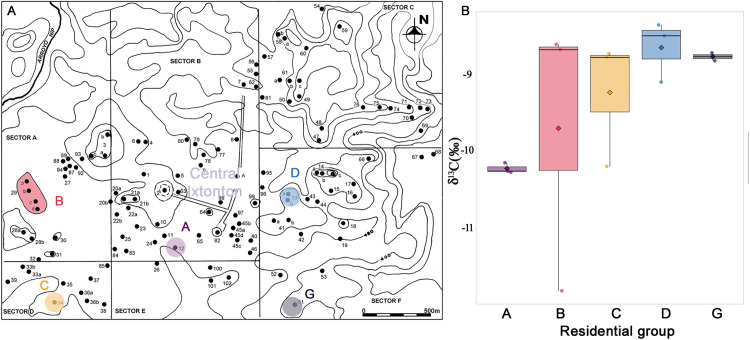
A. Map of Ixtonton showing the location of the five residential complexes (modified from [23]). **B.** Boxplot of δ^13^C_coll_ values by residential complex in Ixtonton.

Within urban Ixtonton, arguably the largest and most politically significant center in southeastern Petén, subtle variation follows residential trends. Individuals with more negative δ^13^C_coll_ values are associated with certain residential groups (Complexes A-C) (Fig 5A, 5B), which may represent households engaged in mixed economic activities, such as various scales of agriculture and different craft production practices. Archaeological evidence from these units, including stonemasonry, textile production, and the manufacture of bone and clay artifacts (Fig 6A; [58]) supports this interpretation. By contrast, individuals from Complexes D and G display higher δ^13^C_coll_ values, which is consistent with higher and more regular maize consumption (Fig 5B). Their proximity to Ixtonton’s central precinct and the presence of distinctive mortuary offerings, such as an incised Torres-type vessel in Burial 94 (Fig 6B; [59]), suggest that these residential areas may have occupied an intermediate social or economic position with greater access to, or reliance on, maize-fed animal products (S2 Table, S1 File).

Although no statistically significant differences were detected between individuals from the Maya Mountains and humid savanna subregions in southeastern Petén, subtle distributions are apparent. Diets in the mountainous zone appear to be more strongly maize-based, while those in the savanna include slightly higher-trophic animal protein. These patterns may reflect the availability of freshwater resources such as fishes, snails, and reptiles in the savannas’ rivers and lakes [60]. Collectively, these patterns highlight the agricultural capacity of southeastern Petén communities and their flexibility in supplementing maize-based subsistence with a range of local resources.

### Diet profile of the southeastern Petén population

#### Age-related dietary patterns.

**Fig 7:**
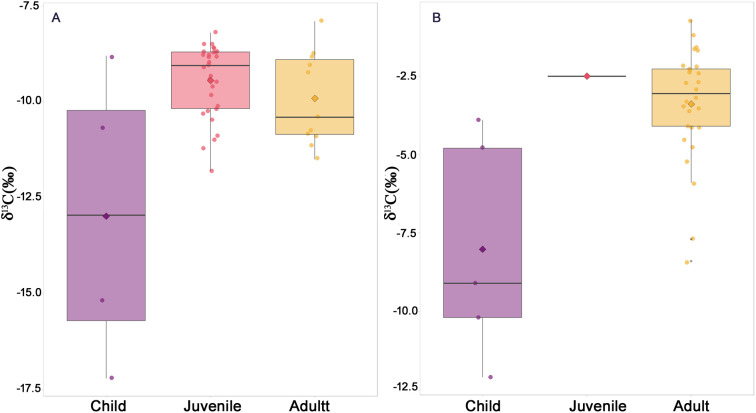
Boxplots of A. δ^13^C_coll_ and B. δ^13^C_enam_ values of southeastern Petén individuals according to age. (Child: 0–10, Juvenile:10–25, Adult: 25–55 years).

Collagen and bioapatite isotopic data reveal a clear age-related shift in diet, with maize consumption increasing from childhood into adulthood. Children (0–5 years) display more variable and, on average, significantly lower δ^13^C_coll_ values than juveniles and adults (S3 and S7 Tables, S1 File; Fig 7), indicating reduced intake of C_4_-derived protein. This variability likely reflects the combined bias of breastfeeding during first infancy and beyond, fat routing, and the gradual introduction of complementary foods. Because maternal diets were strongly maize-based, nursing infants would have inherited elevated δ^13^C_coll_ from the protein and carbohydrate components of breast milk. However, the lipid-rich composition of human milk (naturally ^13^C-depleted relative to other macronutrients) can lead to slightly lower δ^13^C_coll_ values in infant tissues (or tissues formed before and/or during weaning) despite continued trophic enrichment in δ^15^N [61,62]. Consequently, the relatively low δ^13^C_coll_ values observed in some children from southeastern Petén likely reflect both metabolic routing of ^13^C-depleted milk fats and partial reliance on non-maize complementary foods.

Ethnographic and archaeological data from other Maya centers, such as Kaminaljuyú [21], suggest that maize-based gruels (*atole*) were typical weaning foods introduced around the ages of 3–4 [63]. Because isotopic equilibrium in body tissues occurs after this transition [64], elevated δ^15^N values observed in infants, such as in Burial 137C from Copojá, most likely represent trophic enrichment from breastfeeding rather than distinct dietary sources (Fig 7). The relatively low δ^13^C_coll_ value of this individual could suggest either maternal dietary variation or a combination of lipid routing and non-maize weaning foods. Health stress or malnutrition may have also contributed to these isotopic signatures, consistent with broader bioarchaeological indicators of frailty among some adults.

Overall, isotopic evidence indicates that children in southeastern Petén transitioned from mixed or C_3_-influenced weaning diets towards increasingly maize-dominant adult diets. This progression mirrors both nutritional and social maturation processes recognized in Classic Maya society, where the passage from infancy to *ch’ok* (youth) marked entry into a productive social role [65]. Thus, the isotopic shift with age not only reflects physiological development but also embodies a cultural trajectory that links maize consumption with personhood and identity.

#### Sex.

Collagen and bioapatite isotopic data indicate together that males and females in southeastern Petén had broadly similar diets, with no statistically significant differences in δ^13^C_coll_ and δ^15^N values (S7 Table, S1 File). This suggests that both sexes had access to comparable sources of dietary protein and total caloric intake, reflecting a shared subsistence base overall within Classic-period households. Slight trends towards higher δ^13^C_coll_ values (S3 Table, S1 File) among males indicate marginally greater consumption of maize-based protein, consistent with patterns observed at other Classic period sites, such as Pacbitun [66], the Pasión River region [17], and Xcambó in the Northern Lowlands [67]. However, these differences remain within the range of individual variation and are not so divergent as to indicate any formal dietary segregation. Nearly identical δ^15^N values for males and females further support this interpretation, suggesting similar trophic level protein sources and broadly equal access to animal foods. This isotopic parity contrasts with patterns documented elsewhere in the Lowlands, such as Gerry’s [68] finding of more ^15^N-enriched values in males from a large combined dataset of Central and Southeastern sites. In southeastern Petén, the lack of significant sex-based differentiation suggests that food access and consumption were not socially gendered so much as primarily structured at the household level, which is consistent with archaeological evidence of mixed-gender economic activity and cooperative subsistence within domestic units and *milpa* agriculture.

### Diet and status

To investigate whether social stratification influenced dietary patterns between the political entities of southeastern Petén, three contextual variables were examined as potential proxies for status: burial mode, burial type, and rank assignments based on the status scale [27].

#### Burial mode.

**Fig 8:**
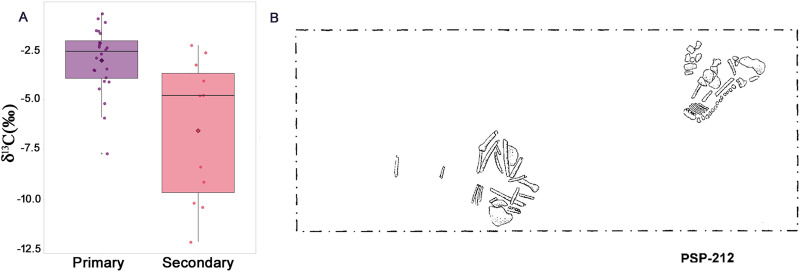
A. Boxplot of δ^13^C_enam_ values of southeastern Petén individuals according to burial mode. **B.** Burial 212 from Calzada Mopán representing a multiple, simple deposition at the foot of the front wall of Structure 1, Group 71. Drawing from Aguirre [72] with institutional copyright by the Archaeological Atlas of Guatemala project coordinated by Mara Reyes and published under Creative Commons Attribution License (CC-BY 4.0).

Primary inhumations dominated the burial sample (84.2% of bone, 71.4% of tooth enamel), with secondary burials comprising the remainder. Primary burials correspond to articulated skeletons [69], while secondary burials range from disarticulated bones [70] to skeletons disarticulated from re-entry [71]. While δ^13^C_coll_ and δ^15^N values did not differ significantly between groups, δ^13^C_enam_ was slightly elevated among secondary burials (Fig 8; S4 and S7 Tables, S1 File). This enrichment appears to be driven mainly by individuals from Burial 212 at Calzada Mopán, a Late Classic multiple deposition of five children and one adult male whose δ^13^C_enam_ values averaged −9.2‰. These values, varying somewhat from the broader sample, may reflect dietary differences related to social identity or geographic origin. Given Calzada Mopán’s prominence during the Terminal Classic and its architectural evidence for migration and cultural interaction [25], this pattern could very well signal incoming populations with distinct dietary practices. This hypothesis will be further evaluated through ongoing ^87^Sr/^86^Sr analyses.

#### Burial type.

Burial construction type varied widely across the study region, from simple interments (n = 29) and cists (n = 37) to a few chamber tombs (n = 3) documented in the urban centers of Ixtonton and Ixtutz [59]. Burial type, including the number and variety of grave goods, has been demonstrated to depend strongly upon an individual’s wealth and status [73]. Interestingly, this study found no significant isotopic differences between the different forms of interment (S7 Table, S1 File), although variability in δ^13^C_coll_ values was the greatest for limestone pit burials, especially those from Copojá and Curucuitz. These sites, previously noted for their distinctive ecological settings and economic activities, may have supported slightly different food economies, with a greater contribution of C_3_ resources (S4 Table, S1 File). By contrast, δ^13^C_enam_ variability among simple burials, again influenced by Calzada Mopán multiple Burial 212, suggests dietary distinctions during childhood that may correspond to mobility or group identity rather than wealth and status alone.

#### Rank assignments.

Most individuals were classified as belonging to low or intermediate rankings (levels 0–1), with only a few assigned to higher status levels (3–4), following criteria laid out by Krejci and Culbert [27] and Tiesler [65], as listed in Price et al. [74]. No statistically significant dietary differences were found between status 0 and 1 (S7 Table, S1 File), though two higher status individuals (level 3) exhibited slightly elevated δ^15^N values consistent with greater access to animal protein and/or higher trophic animal protein (S4 Table, S1 File). These results parallel findings from other regions of the Maya Lowlands, where isotopic distinctions between elites and commoners were minimal or subtle [16]. However, this pattern is not generalized, as Sommerville et al. [20], using a multi-isotopic approach, found more variability in diet among elite individuals than non-elites. As in many other Maya centers, dietary variation among elites may have derived less from nutritional disparities and more from culinary distinctions, as well as the selective consumption of particular foods, preparations, or animal species [6,52] without having a bearing on isotope values. Together, these findings reinforce the view that Classic-period southeastern Petén communities operated within integrated economic systems where subsistence production and consumption were widely shared, despite localized expressions of status and identity in mortuary practice.

### Foodways and biocultural traits

Body practices that remain permanently inscribed in the skeleton, such as cranial modification and dental crafting, provide insights into the intersection of biological, cultural, and social identity among ancient Maya communities [70]. For this study, these traits were examined in the context of isotopically determined dietary profiles to explore how physically embodied social identities were related to foodways among southeastern Petén’s residents.

#### Dental decoration.

Dental decoration was a widespread practice in the region, observed in approximately 63% of adult dental arches, and occurring equally between men and women [75]. Among individuals with preserved enamel, 63% displayed dental inlays, compared with only 33% of the collagen dataset, possibly reflected taphonomic loss and looting as decorated teeth are often taken or removed and curated [24]. Isotopically, individuals with and without dental modification exhibit nearly identical δ^13^C_enam_, δ^13^C_coll_, and δ^15^N values, showing no significant differences between the two groups (S5 and S7 Tables, S1 File). These findings suggest that participation in dental decoration was not associated with differential access to food or distinct dietary regimes. Similar results have been reported for Lamanai [76], as well as across the broader Maya area [6], supporting the interpretation that dental decoration reflected aesthetic or identity-based expressions instead of socioeconomic positions.

#### Cranial modification.

**Fig 9:**
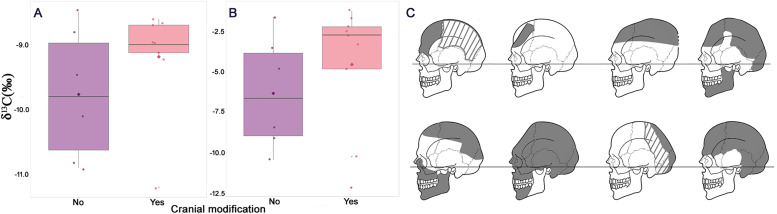
Boxplot of A. δ^13^C_coll_ and B. δ^13^C_enam_ values for individuals with and without cranial deformation. **C.** Reconstruction of cephalic contours in 12 skulls evaluated from Cueva del Cerro, Ixkun, showing mostly no flattening or, where present, slight modeling performed in the cradle. Drawings by Vera Tiesler (LBA/UADY), published under Creative Commons Attribution License (CC-BY 4.0).

Cranial vault shaping was prevalent in southeastern Petén, documented in roughly 80% of evaluable Classic period skulls, most commonly in oblique tabular (34%) and erect (37%) forms [77]. These shapes were achieved through distinct infant modeling techniques: splinting combined with band compression for oblique forms and cradle boards for erect ones [78]. Frequency of such modifications increased towards the Late and Terminal Classic periods, indicating its continued social significance [77]. Among the analyzed individuals, 57% (collagen) and 60% (enamel) showed some form of cranial vault modification, with tabular oblique being the most common. Those with modelled crania showed slightly higher and more consistent δ^13^C_coll_ and δ^13^C_enam_ values (Fig 9), indicating greater reliance on C_4_ resources such as maize or maize-fed fauna. δ^15^N values were, however, similar between groups, suggesting equivalent trophic-level protein intake.

Although cranial modification was independent from social position or status among the Classic and Postclassic Maya [58], variations in regional and local head shapes suggest a lack of standard combined methods or, alternatively, high levels of inter-regional mobility [78]. The small isotopic enrichment observed among individuals with cranial modification may signal subtle differences in food preparation or preferences that favor maize, a food with both nutritional and symbolic significance in Classic Maya cosmology. By contrast, individuals without modification, whose δ^13^C values are generally more negative, may have incorporated higher proportions of C_3_-derived resources into their diets or enjoyed a more diverse intake of carbohydrates and protein (S5 Table, S1 File). However, the fact that most individuals with cranial modifications belong to the Terminal Classic period may introduce a bias into this assessment. Overall, these biocultural markers confirm, once again, that body modification in southeastern Petén was tied more to social traditions than rigid hierarchies, with minor dietary distinctions likely linked to household- or lineage-based food practices rather than broad economic inequality.

### Food practices over time

**Fig 10:**
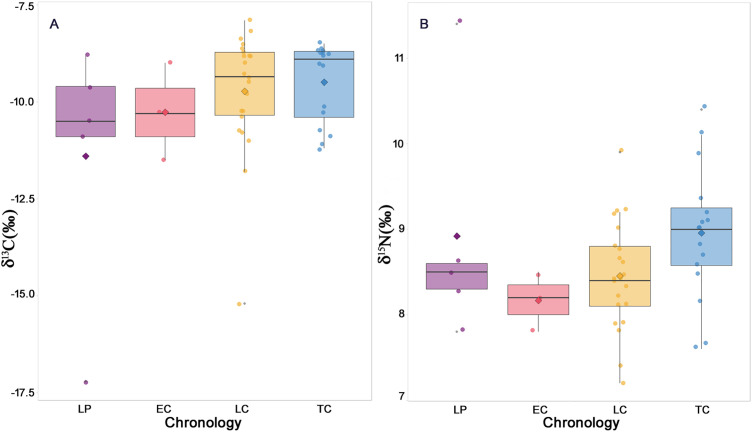
Boxplot of A. δ^13^C_coll_ and B. δ^15^N values in individuals from Southeastern Petén. LP: Late Preclassic, EC: Early Classic, LC: Late Classic, TC: Terminal Classic.

To analyze diachronic changes in the dietary patterns of southeastern Petén’s inhabitants, individuals were grouped by chronological phase. Isotopic evidence indicates a gradual increase in maize consumption from the Late Preclassic period onward (Fig 10A), accompanied by a slight rise in δ^15^N values throughout the Classic and into the Terminal Classic periods (Fig 10B). Although not statistically significant (S7 Table, S1 File), this pattern suggests an intensification of maize production and continued reliance on C_4_-based food systems over time. The corresponding rise in δ^15^N values may reflect greater consumption of animal protein and/or higher trophic animal protein consumption, or environmental factors such as soil aridity, forest clearance, and agricultural stress, which are known to increase plant δ^15^N values [8]. These results suggest that southeastern Petén communities maintained and may have even expanded maize-based subsistence practices during periods when many other regions of the Central Lowlands (North and Central Petén) experienced dietary diversification or a decline in C_4_ intake toward the end of the Classic period. In contrast to coastal sites, the Northern Lowlands, and Copán, where previous research suggests a partial return to higher quantities of C_3_-based resources [6], southeastern Petén populations appear to have maintained a strong commitment to maize cultivation and consumption.

Despite this agricultural and dietary stability, other archaeological evidence documents widespread site abandonment in the region during the Terminal Classic. Interrupted occupation of many centers contrasts with evidence of continued optimal conditions in the region, which boasts numerous river basins and fertile alluvial soils. Estimates from the Dolores Valley suggest that annual maize production would have been sufficient to sustain approximately 1,600 nuclear families, or 8,000 people, indicating that ecological productivity alone cannot account for depopulation [49]. Social or political reorganization, potentially linked to migration and regional realignment, may have played a more substantial role than agricultural potential in the demographic decline during this time.

Continued activity into the Early Postclassic is evident at several activity nuclei, including Ixtonton and Sacul in the Maya Mountains, Calzada Mopán and Ucanal in the middle Mopan River basin, and El Chal and El Muxanal in the humid savanna zone [50]. Interestingly, these early Postclassic centers show the greatest variability in δ^13^C values within the dataset, which suggests flexible or diversified subsistence strategies. This variation may indicate an adaptive response to political and/or environmental instability, which supports the interpretation that post-collapse communities adopted sustainable and resilient approaches to food production and management [16]. In summary, isotopic evidence from southeastern Petén demonstrates continuity instead of disruption in maize reliance extending through the Classic period. This was followed by a period of dietary diversification among the few populations that persisted into the Postclassic. These diachronic trends underscore the enduring importance of maize as a dietary and cultural staple as well as Maya communities’ adaptability to environmental and sociopolitical changes.

## Conclusions

Over the last three decades, stable isotope analysis has become an increasingly important tool for reconstructing ancient diets; however, its interpretive power hinges on robust integration within archaeological and bioarchaeological contexts. In this study, we analyzed isotopic data from human bone collagen and dental enamel alongside detailed archaeological and biovital data to better understand ancient foodways in the southeastern Petén area and its sociocultural regional undercurrents. This is the first regional dataset undertaken at this scale for this still understudied part of the Maya Lowlands. By combining biological and contextual datasets, we have attempted to outline a comprehensive dietary and cultural profile for populations that once inhabited this environmentally diverse landscape.

The isotopic evidence demonstrates that subsistence strategies in southeastern Petén were comparable to other Central Lowlands sites in their reliance on C_4_-based foods, specifically maize. Agricultural activity was a defining feature of the region’s segmentary political organization system, which sustained local populations and enabling the distribution of resources among interconnected political entities. Despite the overall dietary homogeneity, there are some trends that emerged reflecting ecological and socioeconomic variability. For instance, individuals from Curucuitz and residential complex C at Ixtonton; show isotopic values consistent with distinct local economies, whereas the inhabitants of El Chal appear to have incorporated broader resource use, which was likely influenced by its unique savanna environment.

In this region, age related isotopic differences indicate that children were not primarily supplemented with maize products, unlike patterns reported elsewhere in the Maya area. Instead, complementary feeding during the first five years of life seems to have relied on a mixture of C_3_-derived resources and possibly animal protein, with maize consumption increasing progressively from childhood through adulthood. Evidence of a nonlocal or multiethnic presence could account for this and is illustrated by secondary Burial 212 from Calzada Mopán, whose isotopic values suggest a childhood diet with higher proportions of C_3_ resources than that of the local population.

Biocultural traits like cranial modifications show slight but consistent enrichment, which could reflect lineage-based food traditions or the migratory incorporation of some individuals with different cultural affiliations. While this is speculative, forthcoming ^87^Sr/^86^Sr analysis will test these preliminary hypotheses more directly. Temporally, the stability of isotopic signatures across Classic subperiods points to long-term and sustained continuity in subsistence practices, with a slight rise in δ^15^N values during the Terminal Classic, possibly suggesting either increased consumption of higher-trophic-level fauna or environmental shifts in baseline values due to intensified maize agriculture and ecological stress. Importantly, several centers in southeastern Petén persisted into the Postclassic, and these were precisely the ones with more diverse isotopic profiles, implying a flexible and sustainable set of subsistence strategies that may have enhanced their resilience during the broader societal transformations associated with the Maya collapse.

In closing, isotopic and contextual evidence from southeastern Petén portrays a population that balanced agricultural intensification with local ecological adaptations. Maize remained life’s nutritional and symbolic cornerstone; however, its consumption was embedded in flexible, community-specific food systems that endured social and environmental upheaval. Our study underscores the significance of integrating isotopic, archaeological, and biocultural datasets to elucidate how ancient populations navigated subsistence, identity, and resilience within one of the most dynamic environmental and cultural landscapes of the ancient Maya world.

## Supporting information

S1 File Statistical analyses of the collagen and enamel stable isotope values in this study. (DOCX)
