# Supplementary material for: Profiling the diets of Classic Maya communities living in Southeastern Petén through stable isotope analysis
Source: PLoS One. 2026 Jul 22;21(7):e0353029. doi: 10.1371/journal.pone.0353029 (PMC13390858; doi:10.1371/journal.pone.0353029)
Supplement: S1 File — (DOCX) [file pone.0353029.s001.docx]

**Supplementary material**

**S1 Table. Summary and Kruskal-Wallis statistics of δ^13^C and δ^15^N collagen isotopes from southeastern Petén and Classic Central Lowland sites.**

|  | Isotopic system | Mean  (‰) | Median  (‰) | Min  (‰) | Max  (‰) | *p^1^* | *Data from* |
| --- | --- | --- | --- | --- | --- | --- | --- |
| *Central Lowlands* |  |  |  |  |  |  |  |
| Southeastern Petén | δ^13^C | -9.8 | -9.3 | -17.2 | -7.9 |  | This study |
|  | δ^15^N | 8.6 | 8.6. | 7.2 | 11.4 |  |  |
| Aguateca | δ^13^C | -9.6 | -9.3 | -11.0 | -8.8 | 1.0000 | [14] |
| (n=8) | δ^15^N | 9.5 | 9.6 | 7.9 | 10.7 | 1.0000 |  |
| Altar de Sacrificios | δ^13^C | -9.0 | -8.8 | -13.0 | -6.5 | 1.0000 | [14] |
| (n=21) | δ^15^N | 8.8 | 8.7 | 7.7 | 10.5 | 1.0000 |  |
| Calakmul | δ^13^C | -10.7 | -10.2 | -14.5 | -7.8 | 1.0000 | [28] |
| (n=7) | δ^15^N | 11.3 | 11.6 | 9.5 | 13.1 | **0.0000** |  |
| Caracol | δ^13^C | -9.9 | -9.3 | -13.3 | -8.3 | 1.0000 | [30] |
| (n=21) | δ^15^N | 9.6 | 9.5 | 8.5 | 11.2 | **0.0098** |  |
| Dos Hombres | δ^13^C | -13.8 | -13.5 | -21.9 | -9.3 | **0.0002** | [29] |
| (n=18) | δ^15^N | 8.6 | 9.3 | 2.5 | 10.6 | 1.0000 |  |
| Dos Pilas | δ^13^C | -10.0 | -9.0 | -25.0 | -7.1 | 1.0000 | [14] |
| (n=34) | δ^15^N | 10.2 | 10.0 | 7.50 | 16.5 | 1.0000 |  |
| El Perú Waka | δ^13^C | -9.8 | -9.8 | -13.9 | -7.4 | 1.0000 | [31] |
| (n=22) | δ^15^N | 10.6 | 10.7 | 8.6 | 13.3 | 1.0000 |  |
| La Corona | δ^13^C | -9.6 | -9.3 | -11.4 | -8.0 | **0.0000** | [31] |
| (n=12) | δ^15^N | 10.6 | 10.9 | 9.4 | 11.9 | 1.0000 |  |
| La Milpa | δ^13^C | -13.6 | -12.8 | -17.2 | -12.1 | 1.0000 | [29] |
| (n=6) | δ^15^N | 10.0 | 10.2 | 9.1 | 10.5 | 1.0000 |  |
| Lamanai | δ^13^C | -13.6 | -13.6 | -17.2 | -10.6 | **0.0001** | [18] |
| (n=16) | δ^15^N | 10.4 | 10.4 | 9.4 | 13.2 | **0.0000** |  |
| Piedras Negras | δ^13^C | -9.2 | -9.0 | -11.5 | -7.5 | 1.0000 | [13] |
| (n=46) | δ^15^N | 8.7 | 8.6 | 7.6 | 11.5 | 1.0000 |  |
| Seibal | δ^13^C | -9.2 | -8.7 | -13.1 | -6.6 | **0.0085** | [14] |
| (n=53) | δ^15^N | 9.4 | 9.4 | 7.2 | 12.2 | 0.8936 |  |
| Tikal | δ^13^C | -9.6 | -9.3 | -12.8 | -7.7 | 1.0000 | [16] |
| (n=18) | δ^15^N | 9.9 | 9.6 | 7.9 | 13.1 | 1.0000 |  |

^1^Comparison vs. southeastern Petén (Bonferroni adjustment).

**S2 Table.** **Summary and Kruskal-Wallis statistics of δ^13^C collagen isotopes according to carbon groups from southeastern Petén sites, Ixtonton residential complexes, and geographic subregions.**

|  | Mean  (‰) | Median  (‰) | Min  (‰) | Max  (‰) | Trend | *p* |
| --- | --- | --- | --- | --- | --- | --- |
| *Southeastern Petén sites* |  |  |  |  |  |  |
| Low δ^13^C (n=18) | -10.7 | -10.7 | -11.8 | -9.8 | Low δ^13^C< High δ^13^C | **0.000017** |
| High δ^13^C (n=27) | -8.8 | -8.8 | -9.6 | -7.9 |  |  |
|  |  |  |  |  |  |  |
| *Ixtonton residential complexes* |  |  |  |  |  |  |
| A (n=3) | -10.2 | -10.3 | -10.3 | -10.1 | ≈ | >0.1 |
| B (n=3) | -9.7 | -8.7 | -11.8 | -8.6 |  |  |
| C (n=3) | -9.2 | -8.8 | -10.2 | -8.7 |  |  |
| D (n=3) | -8.7 | -8.5 | -9.1 | -8.4 |  |  |
| G (n=2) | -8.8 | -8.8 | -8.8 | -8.7 |  |  |
| *Geographic subregions* |  |  |  |  |  |  |
| Maya mountains (n=36) | -9.6 | -9.2 | -11.8 | -7.9 | Maya Mountains≈ Humid Savannah | 0.1056 |
| Humid savanna (n=7) | -11.5 | -10.5 | -17.2 | -8.6 |  |  |

**S3 Table. Summary statistics of δ^13^C and δ^15^N collagen, and δ^13^C enamel isotopes according to age-at-death and sex.**

|  | Fraction | n | Isotopic system | Mean (‰) | Median  (‰) | Min (‰) | Max (‰) |
| --- | --- | --- | --- | --- | --- | --- | --- |
| *Age* |  |  |  |  |  |  |  |
| Child | Collagen | 4 | δ^13^C | -13.0 | -13.0 | -17.2 | -8.8 |
|  |  |  | δ^15^N | 9.8 | 9.4 | 8.8 | 11.4 |
|  | Enamel apatite | 5 | δ^13^C | -8.0 | -9.1 | -12.1 | -3.9 |
|  |  |  |  |  |  |  |  |
| Juvenile | Collagen | 30 | δ^13^C | -9.4 | -9.1 | -11.8 | -8.2 |
|  |  |  | δ^15^N | 8.6 | 8.5 | 7.4 | 10.1 |
|  | Enamel apatite | 1 | δ^13^C | -2.5 | -2.5 | -2.5 | -2.5 |
|  |  |  |  |  |  |  |  |
| Adult | Collagen | 11 | δ^13^C | -9.9 | -10.4 | -11.5 | -7.9 |
|  |  |  | δ^15^N | 8.5 | 8.3 | 7.2 | 10.4 |
|  | Enamel apatite | 28 | δ^13^C | -3.4 | -3.1 | -8.4 | -0.7 |
| *Sex* |  |  |  |  |  |  |  |
| Males | Collagen | 12 | δ^13^C | -9.2 | -8.8 | -10.9 | -8.2 |
|  |  |  | δ^15^N | 8.6 | 8.6 | 7.8 | 9.4 |
|  | Enamel apatite | 10 | δ^13^C | -3.5 | -2.4 | -7.7 | -2.2 |
|  |  |  |  |  |  |  |  |
| Females | Collagen | 8 | δ^13^C | -10.2 | -10.4 | -11.1 | -9.0 |
|  |  |  | δ^15^N | 8.2 | 8.0 | 7.2 | 9.1 |
|  | Enamel apatite | 7 | δ^13^C | -3.8 | -3.3 | -8.4 | -1.2 |

**S4 Table. Summary statistics of δ^13^C and δ^15^N collagen, and δ^13^C enamel isotopes according to burial mode, burial type, and status (modeled after Krejci and Culbert [25]).**

|  | Fraction | n | Isotopic system | Mean (‰) | Median  (‰) | Min (‰) | Max (‰) |
| --- | --- | --- | --- | --- | --- | --- | --- |
| *Burial mode* |  |  |  |  |  |  |  |
| Primary | Collagen | 32 | δ^13^C | -9.9 | -9.2 | -17.2 | -7.9 |
|  |  |  | δ^15^N | 8.7 | 8.6 | 7.4 | 11.4 |
|  | Enamel apatite | 24 | δ^13^C | -3.06 | -2.6 | -7.7 | -0.7 |
|  |  |  |  |  |  |  |  |
| Secondary | Collagen | 6 | δ^13^C | -9.6 | -9.4 | -11.2 | -8.7 |
|  |  |  | δ^15^N | 9.2 | 9.1 | 7.8 | 10.4 |
|  | Enamel apatite | 11 | δ^13^C | -6.56 | -4.8 | -12.1 | -2.3 |
| *Burial type* |  |  |  |  |  |  |  |
| Simple | Collagen | 5 | δ^13^C | -10.6 | -10.1 | -15.2 | -8.7 |
|  |  |  | δ^15^N | 9.1 | 9.1 | 8.8 | 9.4 |
|  | Enamel apatite | 9 | δ^13^C | -6.76 | -8.4 | -12.1 | -1.6 |
|  |  |  |  |  |  |  |  |
| Pit | Collagen | 3 | δ^13^C | -9.0 | -9.0 | -9.3 | -8.6 |
|  |  |  | δ^15^N | 8.5 | 8.5 | 8.5 | 8.5 |
|  | Enamel apatite | 6 | δ^13^C | -3.2 | -3.3 | -4.8 | -1.2 |
|  |  |  |  |  |  |  |  |
| Limestone carved pit | Collagen | 4 | δ^13^C | -11.9 | -10.9 | -17.2 | -8.5 |
|  |  |  | δ^15^N | 9.5 | 9.5 | 7.6 | 11.4 |
|  | Enamel apatite | 2 | δ^13^C | -3.05 | -3.05 | -4.5 | -1.6 |
|  |  |  |  |  |  |  |  |
| Cist | Collagen | 22 | δ^13^C | -9.6 | -9.2 | -11.8 | -7.9 |
|  |  |  | δ^15^N | 8.6 | 8.6 | 7.2 | 10.4 |
|  | Enamel apatite | 15 | δ^13^C | -3.47 | -2.9 | -7.7 | -1 |
|  |  |  |  |  |  |  |  |
|  |  |  |  |  |  |  |  |
| Chamber^1^ | Enamel apatite | 3 | δ^13^C | -2.5 | -2.7 | -4.1 | -0.7 |
| Status^2^ |  |  |  |  |  |  |  |
| 0 | Collagen | 12 | δ^13^C | -10.2 | -9.6 | -15.2 | -8.6 |
|  |  |  | δ^15^N | 8.7 | 8.5 | 7.2 | 9.9 |
|  | Enamel apatite | 6 | δ^13^C | -2.8 | -2.9 | -3.9 | -1.2 |
|  |  |  |  |  |  |  |  |
| 1 | Collagen | 28 | δ^13^C | -9.6 | -9.2 | -11.5 | -7.9 |
|  |  |  | δ^15^N | 8.6 | 8.6 | 7.4 | 10.4 |
|  | Enamel apatite | 15 | δ^13^C | -3.4 | -3.3 | -5.9 | -1.6 |
|  |  |  |  |  |  |  |  |
| 2 | Collagen | 3 | δ^13^C | -11.7 | -9.3 | -17.2 | -8.7 |
|  |  |  | δ^15^N | 9.2 | 8.4 | 7.7 | 11.4 |
|  | Enamel apatite | NID | δ^13^C |  |  |  |  |
|  |  |  |  |  |  |  |  |
| 3 | Collagen | NID | δ^13^C |  |  |  |  |
|  |  |  | δ^15^N |  |  |  |  |
|  | Enamel apatite | 2 | δ^13^C | -2.0 | -2.0 | -2.9 | -1.0 |
|  |  |  |  |  |  |  |  |

^1^: No chamber burials were found in the collagen set of individuals.

^2^: Statistical comparisons were performed only for individuals assigned as status ‘0’ and ‘1’ according to their number of samples.

**S5 Table. Summary statistics of δ^13^C and δ^15^N collagen, and δ^13^C enamel isotopes according to dental decoration and cranial deformation.**

|  | Fraction | n | Isotopic system | Mean (‰) | Median  (‰) | Min (‰) | Max (‰) |
| --- | --- | --- | --- | --- | --- | --- | --- |
| *Dental decoration* |  |  |  |  |  |  |  |
| Absent | Collagen | 42 | δ^13^C | -9.9 | -9.3 | -17.2 | -7.9 |
|  |  |  | δ^15^N | 8.6 | 8.5 | 7.2 | 11.4 |
|  | Enamel apatite | 21 | δ^13^C | -4.9 | -3.9 | -12.1 | -0.7 |
|  |  |  |  |  |  |  |  |
| Present | Collagen | 5 | δ^13^C | -9.4 | -9.1 | -10.9 | -8.7 |
|  |  |  | δ^15^N | 8.9 | 8.8 | 8.2 | 10.4 |
|  | Enamel apatite | 13 | δ^13^C | -3.0 | -2.7 | -5.9 | -1.6 |
| *Cranial deformation* |  |  |  |  |  |  |  |
| Absent | Collagen | 40 | δ^13^C | -9.7 | -9.5 | -15.2 | -7.9 |
|  |  |  | δ^15^N | 8.5 | 8.5 | 7.2 | 10.1 |
|  | Enamel apatite | 29 | δ^13^C | -4.3 | -3.5 | -10.4 | -4.3 |
|  |  |  |  |  |  |  |  |
| Present | Collagen | 8 | δ^13^C | -10.3 | -9.1 | -17.2 | -8.7 |
|  |  |  | δ^15^N | 9.4 | 9.0 | 8.2 | 11.4 |
|  | Enamel apatite | 4 | δ^13^C | -4.3 | -2.0 | -12.1 | -1.2 |

**S6 Table. Summary statistics of δ^13^C and δ^15^N collagen, and δ^13^C enamel isotopes according to Classic subperiods.**

|  | Fraction | n | Isotopic system | Mean (‰) | Median  (‰) | Min (‰) | Max (‰) |
| --- | --- | --- | --- | --- | --- | --- | --- |
| *Chronology* |  |  |  |  |  |  |  |
| Early Classic | Collagen | 4 | δ^13^C | -9.9 | -9.6 | -11.5 | -8.8 |
|  |  |  | δ^15^N | 8.4 | 8.3 | 7.8 | 9.2 |
|  | Enamel apatite | 5 | δ^13^C | -3.3 | -3.3 | -4.1 | -2.4 |
|  |  |  |  |  |  |  |  |
| Late Classic | Collagen | 23 | δ^13^C | -9.7 | -9.3 | -15.2 | -7.9 |
|  |  |  | δ^15^N | 8.4 | 8.4 | 7.2 | 9.2 |
|  | Enamel apatite | 12 | δ^13^C | -3.1 | -2.4 | -7.7 | -0.7 |
|  |  |  |  |  |  |  |  |
| Terminal Classic | Collagen | 15 | δ^13^C | -9.5 | -9.0 | -11.2 | -8.5 |
|  |  |  | δ^15^N | 9.0 | 9.0 | 7.6 | 10.4 |
|  | Enamel apatite | 18 | δ^13^C | -5.1 | -4.3 | -12.1 | -1.0 |

**S7 Table. Kruskal-Wallis statistics of δ^13^C and δ^15^N collagen, and δ^13^C enamel values according to age-at-death, sex, burial mode, burial type, status, dental decoration, and cranial modification, and ANOVA statistics from stable isotope data according to Classic subperiods.**

|  |  | *p* |  |
| --- | --- | --- | --- |
| ***Age*** | **δ^13^C_coll_** | **δ^15^N_coll_** | **δ^13^C_enam_** |
| Child – Juvenile | 0.1261 | 0.1167 | 0.3374 |
| Child – Adult | 0.8233 | **0.0729** | **0.0136** |
| Juvenile – Adult | 0.6233 | 1.0000 | 1.0000 |
|  |  |  |  |
| ***Sex*** |  |  |  |
| Males - Females | **0.0185** | 0.1890 | 0.4059 |
|  |  |  |  |
| ***Burial mode*** |  |  |  |
| Primary -secondary | 0.4522 | 0.0483 | 0.0014 |
|  |  |  |  |
| ***Burial type*** |  |  |  |
| Cist – Limestone Pit | 1.0000 | 0.6436 | 1.0000 |
| Cist - Pit | 1.0000 | 1.0000 | 1.0000 |
| Limestone Pit – Pit | 0.9257 | 0.6649 | 1.0000 |
| Cist -Simple | 1.0000 | 0.3577 | 0.5137 |
| Limestone Pit – Simple | 1.0000 | 1.0000 | 1.0000 |
| Pit - Simple | 1.0000 | 0.4841 | 1.0000 |
| Chamber – Cist | ^1^ | ^1^ | 1.0000 |
| Chamber - Limestone | ^1^ | ^1^ | 1.0000 |
| Chamber -Pit | ^1^ | ^1^ | 1.0000 |
| Chamber - Simple | ^1^ | ^1^ | 0.5718 |
|  |  |  |  |
| ***Status*** |  |  |  |
| ‘0’ – ‘1’ | 1.0000 | 1.0000 | 1.0000 |
| ‘0’ – ‘2’ | 1.0000 | 1.0000 | ^2^ |
| ‘1’ – ‘2’ | 1.0000 | 1.0000 | ^2^ |
|  |  |  |  |
| ***Dental decoration*** |  |  |  |
| Present -Absent | 0.9511 | 0.4612 | 0.5158 |
|  |  |  |  |
| ***Cranial modification*** |  |  |  |
| Present -Absent | 0.3651 | 0.6048 | 0.3160 |
|  |  |  |  |
| ***Chronology*** |  |  |  |
| Late Preclassic – Early Classic | 1.0000 | 1.0000 | 1.0000 |
| Late Preclassic – Late Classic | 0.8125 | 1.0000 | 1.0000 |
| Late Preclassic – Terminal Classic | 0.7101 | 1.0000 | 1.0000 |
| Early – Late Classic | 1.0000 | 1.0000 | 1.0000 |
| Early – Terminal Classic | 1.0000 | 0.3465 | 1.0000 |
| Late – Terminal Classic | 1.0000 | 0.2680 | 0.1751 |

^1^: No chamber burials were found in the collagen set of individuals.

^2^: No individuals assigned as status 2 were found in the enamel set.
